# Efficacy and safety of wound infiltration modalities for postoperative pain management after cesarean section: a systematic review and network meta-analysis protocol

**DOI:** 10.1186/s13643-022-02068-2

**Published:** 2022-09-07

**Authors:** Semagn Mekonnen Abate, Getachew Mergia, Solomon Nega, Bivash Basu, Moges Tadesse

**Affiliations:** 1 Department of Anesthesiology, College of Health Sciences and Medicine, Dilla University, Dilla, Ethiopia; 2 Departemnt of Obstetrics and Gynecology, College of Health Sciences and Medicine, Dilla University, Dilla, Ethiopia; 3 Departemnt of Internal Medicine, College of Health Sciences and Medicine, Dilla University, Dilla, Ethiopia; 4 School of Public Health, College of Health Sciences and Medicine, Dilla University, Dilla, Ethiopia

**Keywords:** Wound infiltration, Cesarean section, Postoperative pain

## Abstract

**Background:**

Postoperative pain after a cesarean section has negative consequences for the mother during the postoperative period. Over the years, various postoperative pain management strategies have been used following cesarean section. Opioid-based analgesics and landmark approaches have negative side effects, while ultrasound-based regional analgesia necessitates resources and experience, but various wound infiltration adjuvants are innovative with few side effects and are simple to use. The efficacy and safety of each adjuvant, however, are unknown and require further investigation.

**Objective:**

This network meta-analysis is intended to provide the most effective wound infiltration drugs for postoperative management after cesarean section.

**Method:**

A comprehensive search will be conducted in PubMed/MEDLINE, Cochrane Library, Science Direct, CINHAL, and LILACS without date and language restrictions. All randomized trials comparing the effectiveness of wound infiltration drugs for postoperative pain management after cesarean section will be included. Data extraction will be conducted independently by two authors. The quality of studies will be evaluated using the Cochrane risk of bias tool, and the overall quality of the evidence will be determined by GRADEpro software.

**Discussion:**

The rate of postoperative acute and chronic pain is very high which has a huge impact on the mother, family, healthcare practitioners, and healthcare delivery. It is a basic human right to give every patient with postoperative pain treatment that is realistic in terms of resources, technique, cost, and adverse event profile.

**Systematic review registration:**

PROSPERO CRD42021268774

**Supplementary Information:**

The online version contains supplementary material available at 10.1186/s13643-022-02068-2.

## Introduction

### Description of the condition

Cesarean section rates have increased globally over the last three decades, particularly in developed countries [1–5]. According to a World Health Organization report, more than 18 million cesarean sections are performed worldwide each year [4]. However, over 6 million cesarean sections were performed unnecessarily, primarily in the middle- and high-income countries, with China and Brazil accounting for half of all unnecessary cesarean sections, which is higher than the recommended cesarean section rates for a country [1, 3, 4].

The increase in the global trend of the cesarean section presents a significant challenge to postoperative pain management globally [6]. Different postoperative pain management modalities have been employed over the years after cesarean section. However, none of them is with postoperative adverse events to the mother. Systemic opioid and non-opioid medications, regional blocks, and local wound infiltration of various local anesthetics and other drugs are among the most commonly used postoperative pain treatment strategies [7–20].

Evidence revealed that individual pain variability is highly influenced by different factors such as pain sensitivity, gender, age, heredity, preoperative anxiety, preoperative pain, history of depressive symptoms, and history of substance use [6, 21–27].

Despite advances in the understanding of the pathophysiology of postoperative pain and the introduction of various postoperative analgesic drugs and modalities, the prevalence of postoperative pain after cesarean section remains high, ranging from 25.5 to 80% [6, 21, 22, 25, 26, 28, 29].

Postoperative pain after a cesarean section has an unfavorable impact on ambulation, breastfeeding, and maternal attachment to their newborn [30]. Furthermore, poorly managed acute postoperative pain is linked to a variety of consequences, including postpartum depression, myocardial infarction, pulmonary infection, decreased gastric motility, nausea, vomiting, impaired immune function, and impaired wound healing [21, 26, 27].

### Description of the intervention

Postoperative wound infiltration techniques after cesarean have been employed due to their convenience of use and feasibility in terms of cost-effectiveness, administration processes, and adverse effects. The commonly used approach was wound infiltration with local anesthetics alone or coupled with adjuvants [8, 13, 17–19, 31–42]. However, recent studies comparing local anesthetics with glucocorticoids, opioids [16, 20, 36, 43–46], ketamine [14, 32, 34, 47–50], nonsteroidal anti-inflammatory agents [51], alpha 2 agonists [8, 52], and magnesium sulphate [37, 53, 54] are emerging.

### How the intervention might work

Enhanced recovery after caesarean section recommends multimodal analgesics with minimum side effects, various sites of action, and opioid sparing analgesia as much as feasible for postoperative pain management after caesarean Sect. [55, 56]. In this regard, a number of techniques and drugs have been employed over the years for postoperative pain management after a cesarean section including epidural analgesia, Transvers abdominis plan block, Illioinguinal and Illiohypogastric nerve block, quadratus lumborum block, and abdominal wound infiltration with different drugs [7, 10, 57, 58]. This network meta-analysis, however, aims to investigate the efficacy and safety of several wound infiltration medications for postoperative pain relief following a cesarean section. Local anesthetics are known to be injected into localized wounds where they block the sodium channel to stop the propagation of action potentials and pain perception [59–61]. Despite disagreements regarding their effectiveness, various medications like ketamine, dexamethasone, week opioids, selective alpha 2 agonists, and magnesium sulphate are used alone or as adjuvants to local anesthetics. Although the exact mechanism by which each medication reduces pain is unknown, it is believed to be accomplished through inhibiting neuronal transmission, inflammatory mediators, and *N*-methyl-d-aspartate (NMDA) receptors [38, 62–70].

### Why is it important to do this review?

Evidence revealed that the rate of a cesarean section is steadily increasing globally. The magnitude of postoperative pain following cesarean delivery is relatively high, posing a significant challenge to health care providers. Inadequate postoperative care after a cesarean section can lead to deep venous thrombosis, delayed breastfeeding, paralytic ileus, postpartum depression, lung infection, delayed wound healing, longer hospital stays, persistent discomfort, and greater health care expenses. As a result, various postoperative pain management strategies have been used following a cesarean section. Opioid-based analgesics and landmark approaches, on the other hand, have undesirable consequences; regional analgesia with ultrasonography demands resources and experience, whereas wound infiltration treatments are innovative procedures with few side effects and are simple to apply. However, the efficacy and safety of each adjuvant are uncertain and need further investigation with a network meta-analysis.

## Methods

### Protocol and registration

This network meta-analysis will be conducted based on the Preferred Reporting Items for Systematic and Meta-analysis protocol (PRISMA-P) [71]. The network meta-analysis protocol was registered in PROSPERO CRD42021268774 on August 19, 2021.

### Eligibility criteria

#### Types of studies

All randomized controlled trials assessing the efficacy and safety of wound infiltration with ketamine, opioids, alpha 2 agonist, magnesium, steroids, and local anesthetics for postoperative pain management following a cesarean section will be considered. However, observational studies comparing wound infiltration to placebo and other medications must be excluded since they are done in the heterogeneous groups of patients with diverse confounders, which may buffer the effect size of this network meta-analysis. Besides, a comparison of local anesthesia with regional block will be excluded.

#### Types of participants

The American Society of Anesthesiologists physical status classifications (ASA) I and II, term pregnancy, age greater than 18 years scheduled for a cesarean section under spinal anesthesia, will be included, while the rest will be excluded. The inclusion and exclusion criteria were defined by each primary included study.

#### Types of intervention

The treatment group will be parturient allocated to one of the wound infiltration drugs which were as per the included studies, while the parturient allocated to the comparator defined by each included study will be considered as controlled groups.

#### Outcome measures

The primary outcomes of this network meta-analysis protocol will be postoperative pain severity, first analgesic request, total morphine equivalent analgesic consumption, and patient satisfaction, while secondary outcomes will be postoperative nausea and vomiting, sedation, hallucination, dizziness, bleeding, hypotension, hypertension, bruising, infection, and mortality.

### Search strategy

The goal of the search strategy is to find all published and unpublished randomized controlled trials comparing wound infiltration modalities for postoperative pain management after a cesarean section among parturient undergoing a cesarean section under spinal or general anesthesia, regardless of language or date restrictions.

A comprehensive initial search of PubMed/MEDLINE, Cochrane Library, Science Direct, and Latin American and Caribbean Health Sciences Literature (LILACS) will be conducted, followed by an analysis of the text words found in the title/abstract and indexed keywords. A second search will be performed by combining free text words with indexed phrases using Boolean operators. In addition, the third search might be conducted utilizing the reference lists of all papers and publications. Finally, a gray literature search will be conducted using the Google Scholar. EndNote reference manager could be used to remove the duplicates. The remainder will then be considered for inclusion in the systematic review using the PICO technique, which will be carried out in various databases depending on database-specific criteria Mesh terms OR cesarean section OR C-section OR Cesarean delivery AND local anesthetics OR bupivacaine OR Levobupivacaine OR Marcaine OR Lidocaine OR Opioids OR tramadol OR pethidine OR ketamine OR dexamethasone OR steroid OR Glucocorticoid OR Dexmedetomidine OR clonidine OR α2 agonist AND wound infiltration OR subcutaneous infiltration OR abdominal infiltration AND Normal saline OR placebo AND postoperative pain OR analgesia OR toxicity OR adverse effects OR RCT. The detailed search strategies are presented as supplementary material (Supplemental Table 1). The results of the search strategy will be summarized with a Prisma flow chart [72].

### Data extraction

Two independent reviewers will retrieve the data using a customized Microsoft Excel 2013 format, and the differences between the two independent reviewers will be settled by the other two reviewers. Names of authors, country, year of publication, sample size, mean age, treatment and control groups, pain intensity, initial analgesic request, overall analgesic consumption, patient satisfaction, nausea and vomiting, sedation, hallucinations, dizziness, infection at the injection site, and other events will be included in the data extraction template. The risk of bias summary for each included study will be calculated after the data have been entered into the review manager. When appropriate, the pairwise meta-analysis, network meta-analysis, meta-regression, and publication bias, as well as sensitivity analysis, shall be carried out using STATA 16 or R software version 4.1.3.

### Critical appraisal of included studies

The methodological quality of included studies will be assessed by two independent reviewers using the Cochrane handbook Risk of Bias Tool (ROB 2) for systematic reviews of intervention [73], and disagreements will be handled by the other reviewers. Random sequence generation, allocation concealment, blinding of participants and treatment providers, blinding of result assessment, inadequate outcome data, selective outcome reporting, and other bias risks should be assessed. A critical evaluation tool for systematic reviews that contain randomized or non-randomized trials of healthcare interventions, or both, might be used to assess the methodological quality of this systematic review (AMSTAR 2) [73].

#### Random sequence generation

Studies assessed a computer random number generator or a random number table to generate random sequences were classified as having a minimal risk of bias. Aside from using the lottery technique to generate random sequences, an independent adjudicator can also use tossing a coin, shuffling cards, or throwing dice. It is considered an unknown risk of bias if the technique of randomization was not mentioned yet the experiment was nonetheless presented as randomized.

If the allocation sequence was not randomized or simply quasi-randomized, there is a substantial chance of bias.

#### Allocation concealment

The patients are assigned by a central independent unit, an on-site closed computer, or identical-looking numbered sealed envelopes or containers generated by an independent investigator, and allocation concealment is considered low risk. If the study was classed as randomized but the allocation concealment procedure was not specified, there is a low risk of bias, and if the allocation sequence was familiar to the investigators who allocated participants, there is a high risk of bias.

### Blinding of participants and treatment providers

If the participants and the treatment providers were blinded to intervention allocation and this was described in the article, it is considered to be low risk of bias and it was uncertain if the procedure of blinding was insufficiently described. If blinding of participants and the treatment providers was not performed at all, it was taken as a high risk of bias.

#### Blinding of outcome assessment

It is said to have a low risk of bias if the outcome assessors were blinded and this was adequately described, but it is unclear if the outcome assessors in the trial were blinded or the extent of blinding was insufficiently described, and it is said to have a high risk of bias if no blinding or incomplete blinding of outcome assessors was performed.

#### Incomplete outcome data

There were no drop-outs or withdrawals for all outcomes, the numbers and reasons for all withdrawals and drop-outs for all outcomes were clearly stated and could be described as being similar to both groups, or if drop-outs were less than 5%, there is a low risk of bias. If there was insufficient information to assess whether missing data were likely to induce bias on the results, an uncertain risk of bias is assumed. However, a high risk of bias is considered if the results were likely to be skewed by missing data, either because the pattern of drop-outs in the two intervention groups was different or the trial utilized poor strategies to deal with missing data.

#### Selective outcome reporting

A low risk of bias is considered if a protocol was published before or at the time the trial began, and the outcomes specified in the protocol were reported, and uncertain risk of bias is rated if no protocol was published. If the outcomes in the protocol were not reported at all, a high risk of bias is introduced.

#### Other risks of bias

If the study looks to be devoid of additional factors that may lead to bias (such as academic or commercial prejudice), it has a low risk of bias. If the study may or may not be free of additional components that may put it at risk of bias but are not stated, it is referred to as unclear risk of bias.

Other elements in the study that might add bias, such as authors performing studies on the same topic or for profit, could raise the likelihood of bias significantly.

### The overall risk of bias

Overall, the study is said to have a low risk of bias only if all of the bias domains described are classified as low risk of bias and high risk of bias if any of the bias risk domains described above are classified as “unclear” or high risk of bias.

#### Grading the quality of evidence

The overall quality of evidence for the studied outcome will be evaluated using the GRADE system (Grading of Recommendations, Assessment, Development, and Evaluation) [52, 74]. The system incorporates study quality (risk of bias), inconsistency (comparison of effect estimates across studies), indirectness (applicability of the population, intervention, comparator, and outcomes to the clinical decision), and imprecision (certainty of confidence interval) and high probability of publication bias. The overall quality of evidence will be categorized by evaluating and combing the above five parameters for maternal.

### Data analysis

The data will be analyzed using Review Manager Version 3.3.1 software, R statistical software version 3.6.1, and STATA 16 where applicable. The pooled incidence of postoperative pain, the weighted mean difference in pain score, the first analgesic request, and adverse effects such as nausea and vomiting, hallucination, dizziness, sedation, bleeding at injection site, local anesthetic toxicity, hypotension, hypertension, and infection with fixed and random effect models using the restricted maximum likelihood (REML) method where appropriate, but the meta-analysis results will be reported with random effect model if there is substantial heterogeneity between the included studies.

The heterogeneity among the included studies will be checked with forest plot, *χ*^2^ test, *I*^2^ test, and the *p* values. Subgroup analysis will be conducted by the type of intervention, dose range, and types of pain rating scale as postoperative pain usually assessed either with numeric rating scale (NRS) or visual analog scale (VAS). Meta-regression is planned to be conducted with a year of publication, mean age, and sample size. Furthermore, sensitivity analysis might be done to examine the impact of each study on summary effect size by omitting studies one at a time.

Publication bias could be examined with a funnel plot, and the objective diagnostic test will be conducted with Egger’s correlation, Begg’s regression tests, and the Trim and fill method. Network meta-analysis will be performed with the “netmeta” of R version 4.1.3 software to synthesize direct and indirect evidence for efficacy and safety of postoperative pain modalities after cesarean section.

### Data synthesis

#### Narration

The authors want to discuss the sample size, country, intervention, comparator, methodological quality, mean age of participants, baseline clinical factors, primary and secondary outcomes, conclusion, and recommendation of each included study. In addition, the table will summarize the descriptions of the included studies.

#### Pairwise meta-analysis

This systematic review will be conducted in compliance with the updated Cochrane Handbook for Systematic Reviews of Interventions [75]. The meta-analysis will be conducted with review manager 5 [76] to estimate the pooled effect sizes and risk of bias summary while, STATA 16 software [77], and R software version 4.2 [78] will be used for meta-regression, sensitivity analysis, and publication bias analysis where appropriate. We will conduct the meta-analysis with a restricted maximum likelihood (REML) estimator with both random and fixed effect models as recommended by different authors [79, 80]. Substantial heterogeneity among the included studies will be investigated with subgroup analysis and meta-regression, and the final decision to report the finding either narratively or doing the meta-analysis with a random effect model depends on the clinical importance of the outcome [81–84]. Publication bias will be checked with a funnel plot, and the objective diagnostic test will be conducted with Egger’s correlation, Begg’s regression tests, and the trim and fill method.

#### Network meta-analysis

The network meta-analysis will be performed with the “netmeta” of R version 4.1.3 software to synthesize direct and indirect evidence for assessing the efficacy and safety among different wound infiltrative medications (local anesthetics, opioids, ketamine, nonsteroidal anti-inflammatory agents, alpha two agonists, and magnesium sulfate) regimens for postoperative pain management after cesarean section. The inconsistency between direct and indirect comparisons will be assessed by the node splitting method when a loop connecting three arms existed. *P* scores will be used to rank the treatment effects of different wound infiltration regimens which are based on the point estimates and standard errors of the network assessment.

#### The geometry of the network

The function of “forest. Netmeta” of R software version 4.1.3 will be used to draw network plots to describe and present the geometry of different wound infiltration regimens. The nodes and edges will be used to reveal the head-to-head comparisons among interventions.

### Patient and public involvement

No patient involved.

#### Ethics and dissemination

Ethical approval is not required for this study as it is a systematic review and meta-analysis. The final result will be presented to international and national conferences. Besides, the manuscript will be published in a national or international peer-reviewed journal.

## Discussion

This network meta-analysis is planned to investigate wound infiltration postoperative pain management modalities after a cesarean section.

A systematic review and meta-analysis, and randomized controlled trials revealed that systemic opioid-based analgesics, neuraxial analgesia, and locoregional blocks provide better postoperative pain relief after a cesarean Sect. [7, 18, 30, 62–64, 85–89]. However, systemic opioid-based analgesics are associated with several postoperative adverse events including nausea, vomiting, respiratory depression, opioids addiction, and other gastrointestinal complications [88, 90]; neuraxial and thoracoabdominal field block requires resources, expertise, and are also associated with complications including hypotension, high spinal, bradycardia, nerve damage, organ damage, and local anesthetics toxicity [91, 92].

Local wound infiltration techniques using local anesthetics, ketamine, opioids, dexmedetomidine, glucocorticoids, and nonsteroidal anti-inflammatory agents, on the other hand, are feasible due to technical issues, resources, low complication rates, and patient acceptance, despite effectiveness and superiority differences [93].

Evidence revealed that the incidence of postoperative acute and chronic pain is quite high, having a significant influence on the mother, family, healthcare practitioners, and healthcare delivery [21, 26, 27].

## Supplementary Information

**Additional file 1: Supplemental Table 1.** Comprehensive search strategies of different databases as per PICOs criteria.

## Data Availability

Data and material can be available where appropriate.
