# Supplementary material for: Efficacy and safety of wound infiltration modalities for postoperative pain management after cesarean section: a systematic review and network meta-analysis protocol
Source: Syst Rev. 2022 Sep 7;11:194. doi: 10.1186/s13643-022-02068-2 (PMC9450460; doi:10.1186/s13643-022-02068-2)
Supplement: Supplementary file 1 — Additional file 1: Supplemental Table 1. Comprehensive search strategies of different databases as per PICOs criteria. [file 13643_2022_2068_MOESM1_ESM.docx]

# **Supplemental table 1: comprehensive search strategies of different databases as per PICOs criteria**

| Databases | Search details | FILTERS USED |
| --- | --- | --- |
| PubMed/Medline | Search query: (((((((((((((((((((((((((((((((((((((((("cesarean section"[MeSH Terms] OR cesarean section[Text Word]) ) OR (Delivery, Abdominal[MeSH Terms])) OR (caesarean section[MeSH Terms])) OR (cesarean delivery[MeSH Terms])) OR (Abdominal Deliveries[MeSH Terms])) OR (C-Section (OB)[MeSH Terms])) AND (wound infiltration[MeSH Terms])) OR (subcutaneous infiltration[MeSH Terms])) OR (abdominal infiltration[MeSH Terms]) AND (local anesthetics[MeSH Terms])) OR (Levobupivacaine[MeSH Terms])) OR (bupivacaine[MeSH Terms])) OR (marcaine[MeSH Terms])) OR (lidocaine[MeSH Terms])) OR (analgesics, opioid[MeSH Terms])) OR (tramadol[MeSH Terms])) OR (pethidine[MeSH Terms])) OR (morphine[MeSH Terms])) OR (ketamine[MeSH Terms])) OR (dexamethsone[MeSH Terms])) OR (dexmedetomidine[MeSH Terms])) OR (clonidine[MeSH Terms])) OR (magnesium salphate[MeSH Terms])) OR (steriods[MeSH Terms])) OR (glucocorticoids[MeSH Terms])) AND (Normal saline[MeSH Terms])) OR (placebo[MeSH Terms])) AND (pain[MeSH Terms])) OR (analgesia[MeSH Terms])) OR (NRS score[MeSH Terms])) OR (VAS score[MeSH Terms])) OR (analgesic consumption[MeSH Terms])) OR (analgesic request[MeSH Terms])) OR (toxicity[MeSH Terms])) OR (complication[MeSH Terms])) OR (sedation[MeSH Terms])) OR (nausea[MeSH Terms])) OR (vomiting[MeSH Terms])) AND (randomized controlled trial[MeSH Terms])) OR (RCT[MeSH Terms])) | *Free full text, Clinical Trial, Randomized Controlled Trial, Adult: 19+ years, Humans* |
| Cochrane library | ID Search  #1 MeSH descriptor: [Cesarean Section] explode all trees  #2 MeSH descriptor: [Injections, Subcutaneous] explode all trees  #3 MeSH descriptor: [Anesthetics, Local] explode all trees  #4 MeSH descriptor: [Bupivacaine] explode all trees  #5 MeSH descriptor: [Levobupivacaine] explode all trees  #6 MeSH descriptor: [Lidocaine] explode all trees  #7 MeSH descriptor: [Ketamine] explode all trees  #8 MeSH descriptor: [Analgesics, Opioid] explode all trees  #9 MeSH descriptor: [Tramadol] explode all trees  #10 MeSH descriptor: [Morphine] explode all trees  #11 MeSH descriptor: [Dexmedetomidine] explode all trees  #12 MeSH descriptor: [Clonidine] explode all trees  #13 MeSH descriptor: [Steroids] explode all trees  #14 MeSH descriptor: [Dexamethasone] explode all trees  #15 MeSH descriptor: [Magnesium] explode all trees  #16 MeSH descriptor: [Saline Solution] explode all trees  #17 placebo  #18 MeSH descriptor: [Analgesia] explode all trees  #19 MeSH descriptor: [Hallucinations] explode all trees  #20 MeSH descriptor: [Postoperative Complications] explode all trees  #21 MeSH descriptor: [Randomized Controlled Trial] explode all trees  #22 #1 AND #2  #23 #3 8 OR #4 OR #5 OR #6 OR #7 OR #8 OR #9 OR #10 OR #11 OR #12 OR #13 OR #14 OR #15  #24 #16 OR #17  #25 #18 OR #19 OR #20  #26 #21  #27 #22 AND #23 AND #24 AND #25 OR #26 | *Clinical Trial, Randomized Controlled Trial,* |
| SCIENCE DIRECT | The database will be searched with PICOs criteria as cesarean section OR cesarean section OR C-section OR Cesarean delivery AND local anesthetics OR bupivacaine OR Levobupivacaine OR Marcaine OR Lidocaine OR Opioids OR tramadol OR pethidine OR ketamine OR dexamethasone OR steroid OR Glucocorticoid OR Dexmedetomidine OR clonidine OR α2 agonist AND wound infiltration OR subcutaneous infiltration OR abdominal infiltration AND Normal saline OR placebo AND postoperative pain OR analgesia OR toxicity OR adverse effects OR RCT | *Research articles, Clinical Trial, Randomized Controlled Trial* |
| LILACS | The database will be searched with PICOs criteria as cesarean section OR cesarean section OR C-section OR Cesarean delivery AND local anesthetics OR bupivacaine OR Levobupivacaine OR Marcaine OR Lidocaine OR Opioids OR tramadol OR pethidine OR ketamine OR dexamethasone OR steroid OR Glucocorticoid OR Dexmedetomidine OR clonidine OR α2 agonist AND wound infiltration OR subcutaneous infiltration OR abdominal infiltration AND Normal saline OR placebo AND postoperative pain OR analgesia OR toxicity OR adverse effects OR RCT |  |
| Google scholars | The database will be searched with PICOs criteria as cesarean section OR cesarean section OR C-section OR Cesarean delivery AND local anesthetics OR bupivacaine OR Levobupivacaine OR Marcaine OR Lidocaine OR Opioids OR tramadol OR pethidine OR ketamine OR dexamethasone OR steroid OR Glucocorticoid OR Dexmedetomidine OR clonidine OR α2 agonist AND wound infiltration OR subcutaneous infiltration OR abdominal infiltration AND Normal saline OR placebo AND postoperative pain OR analgesia OR toxicity OR adverse effects OR RCT |  |
